# Evaluating Lattice Mechanical Properties for Lightweight Heat-Resistant Load-Bearing Structure Design

**DOI:** 10.3390/ma13214786

**Published:** 2020-10-27

**Authors:** Xinglong Wang, Cheng Wang, Xin Zhou, Di Wang, Mingkang Zhang, Yun Gao, Lei Wang, Peiyu Zhang

**Affiliations:** 1 Science and Technology on Plasma Dynamics Laboratory, Air Force Engineering University, Xi’an 710038, China; wxlnpp@126.com (X.W.); warrant_74@126.com (C.W.); peiyuzhang1128@gmail.com (P.Z.); 2 Basic Department, Air Force Engineering University, Xi’an 710051, China; wanglei.8181@163.com; 3 School of Mechanical & Automotive Engineering, South China University of Technology, Guangzhou 510640, China; mewdlaser@scut.edu.cn (D.W.); 201610100405@mail.scut.edu.cn (M.Z.); 4 Xi’an Aerospace Mechatronics & Intelligent Manufacturing Co., LTD, Xi’an 710100, China; gaoyun031@163.com

**Keywords:** lattice mechanical properties, lattice topology optimization, heat-resistant load-bearing structure, metal lattice structure, triply periodic minimal surface, selective laser melting

## Abstract

Heat-resistant, load-bearing components are common in aircraft, and they have high requirements for lightweight and mechanical performance. Lattice topology optimization can achieve high mechanical properties and obtain lightweight designs. Appropriate lattice selection is crucial when employing the lattice topology optimization method. The mechanical properties of a structure can be optimized by choosing lattice structures suitable for the specific stress environment being endured by the structural components. Metal lattice structures exhibit excellent unidirectional load-bearing performance and the triply periodic minimal surface (TPMS) porous structure can satisfy multi-scale free designs. Both lattice types can provide unique advantages; therefore, we designed three types of metal lattices (body-centered cubic (BCC), BCC with Z-struts (BCCZ), and honeycomb) and three types of TPMS lattices (gyroid, primitive, and I-Wrapped Package (I-WP)) combined with the solid shell. Each was designed with high level of relative density (40%, 50%, 60%, 70%, and 80%), which can be directly used in engineering practice. All test specimens were manufactured by selective laser melting (SLM) technology using Inconel 718 superalloy as the material and underwent static tensile testing. We found that the honeycomb test specimen exhibits the best strength, toughness, and stiffness properties among all structures evaluated, which is especially suitable for the lattice topology optimization design of heat-resistant, unidirectional load-bearing structures within aircraft. Furthermore, we also found an interesting phenomenon that the toughness of the primitive and honeycomb porous test specimens exhibited sudden increases from 70% to 80% and from 50% to 60% relative density, respectively, due to their structural characteristics. According to the range of the exponent value n and the deformation laws of porous structures, we also concluded that a porous structure would exhibit a stretching-dominated deformation behavior when exponent value n < 0.3, a bending-dominated deformation behavior when n > 0.55, and a stretching-bending-dominated deformation behavior when 0.3 < n < 0.55. This study can provide a design basis for selecting an appropriate lattice in lattice topology optimization design.

## 1. Introduction

Lightweight design is a key technical direction for domestic and foreign aerospace development that can improve the maneuverability, flight speed, range, and payload of missile weapons and space vehicles [1]. Earlier lightweight designs of heat-resistant, load-bearing structures were based on the experience of engineers and numerous experiments. Upon determining the basic configuration, intelligent algorithms were employed to optimize the size and shape and reduce the weight [2,3]. However, such designs often take numerous cycles to develop and offer poor weight reduction effects. A successful design contains a lattice structure that is suitable for the specific stress environment the structural component will experience. This can be accomplished by optimizing the lattice topology, which is a method that obtains a complex, solid lattice hybrid structure by selecting the appropriate lattice structure that would fill in the material removal portion of the topologically optimized structure within a certain relative density interval [4]. The development of topology optimization theory and commercial optimization software could provide a new route for designing lightweight structures [5,6]. Meanwhile, lattice structure design and studies on their performance have received extensive attention [7]. Recent research results show that lattice topology optimization helps to design lightweight structures with improved mechanical properties [8]. Reasonable selection of the lattice type and correctly setting the relative density interval of the lattice filling can optimize the mechanical properties of the solid lattice hybrid structure [4,8]. At present, commonly employed lattice types include foam porous structures, metal lattice structures, and triply periodic minimal surface (TPMS) porous structures [9,10,11]. Among them, the metal lattice structure presents good unidirectional load-bearing characteristics [12], while the TPMS porous structure exhibits self-supported characteristics that greatly improves its design freedom [13]. Both lattice types are suitable for the lattice topology optimization design of heat-resistant, unidirectional load-bearing structures.

Series of studies have been conducted on the additive manufacturing of these lattice structures and the relationship between the characteristic variables (e.g., lattice size, relative density, number of unit cell arrays, and loading conditions) and the lattice mechanical properties [14,15,16]. Yang [17] conducted a statistical analysis of the geometric factors affecting the mechanical properties of the gyroid TPMS structure and found that the number of pores had the greatest impact on structural rigidity and strength, followed by the thickness of the surface. Zhang [18] compared the compression modulus, platform stress, and energy absorption capacity of the three types of TPMS sandwich structures (primitive, diamond, and gyroid) and found that the diamond structure exhibited the best mechanical properties. In another study, Li [19] established an analytical model of a 3D re-entrant honeycomb auxetic cellular structure, which provided a convenient and relatively accurate method for predicting auxetic cellular structure performance once the manufacturing related factors were adequately incorporated into the model. Rashid [20] designed two different topologically optimized beams with the Bi-directional Evolutionary Structural Optimization (BESO) algorithm and used them in three-point bending tests. The results show that these topologically optimized beams have better flexural properties and energy absorption characteristics than solid beams. Rashid [21] designed samples with different lattice core geometries and three-point bend tests were performed to study the failure mechanisms of lattice. The result shows that the samples with triangular lattice core structures exhibited better flexural properties compared with other structures. Few studies have used the heat-resistant, load-bearing structure for the background of lightweight design. There were various studies focused on lattice topologies which generated a high porosity up to 90% [22]. The heat resistant load-bearing structure is made of Inconel718 superalloy, and the optimized pore area needs to be filled with lattice structure at medium and high relative density to ensure the mechanical safety margin of the structure. Therefore, there exists no efficient guidance for optimizing the lattice topology of such heat-resistant, load-bearing structures [23].

This study focused on the design, lattice topology optimization, and additive manufacturing of lattice structures that exhibit heat resistance and load bearing characteristics for employment in aircraft. Three types of metal lattices (BCC, BCCZ, and honeycomb) and three types of TPMS structures (gyroid, primitive, and I-WP) combined with the solid shell were designed, all of which possessed high level of relative density (40%, 50%, 60%, 70% and 80%). All test specimens were manufactured from Inconel718 superalloy by selective laser melting (SLM) technology [24,25,26]. Static tensile tests were carried out to study and compare the advantages and disadvantages of the mechanical properties of each porous test specimen filled with lattice structure at different relative densities and to observe any property changes. Micromorphology analysis was performed on the fracture surface of the test specimens using scanning electron microscopy (SEM) and the complex effects of topological design (e.g., strut connections) and micro-scale structure on the mechanical properties of the test specimens were also studied. Our results provide a design basis for selecting the appropriate lattice type for designing and optimizing the topology of heat-resistant, load-bearing structures.

## 2. Design and Experiment

### 2.1. Design of Porous Test Specimen

Metal lattice structures and TPMS porous structures were selected as the candidate unit cells to fill the pore area of the topologically optimized heat-resistant, load-bearing structure. The metal lattice structure design is analogous to the spatial arrangement of crystal material particles (e.g., atoms, ions, and molecules), which is one of the important methods for lightweight design. Throughout this study, three configurations of the metal lattice structure were considered. The BCC lattice structure consists of eight struts radiating from the centers of two adjacent cubes to the four vertices of the common plane. The BCCZ configuration is built over the BCC, where a vertical strut is added between the two central vertices of the BCC. It can be regarded as a reinforced version of the BCC configuration. The honeycomb lattice is composed of two hexagons that are arranged opposite to each other and connected through their vertices by struts. The main, load-bearing struts of the BCCZ and honeycomb structures in the unit cell array must be located along the bearing direction of the test specimen. This is to ensure that the designed unit cell exerts the best, unidirectional mechanical performance.

**Figure 1:**
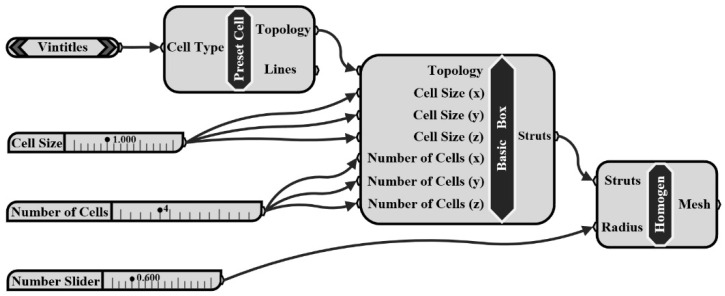
Metal lattice structure unit cell design flow diagram.

The metal lattice structure unit cell was constructed by parametric modeling method. The “Preset Cell” in the flow chart (Figure 1) was used to control the basic configuration of the constructed metal lattice structure. The unit cell size and number of arrays in the x, y, and z directions can be adjusted by dealing with the corresponding values in the “Basic Box”. The “Radius” in “Homogen” controls the radius of the unit cell struts; therefore, unit cells with different volumes can be obtained by inputting different “Radius” values. The acquired model can be intersected by the unit cube volume using a Boolean operation to obtain the unit cell with the desired relative density.

The struts of the metal lattice unit cell are affected by their own weight; therefore, there are design limitations regarding the length-to-diameter ratio and the angle to the horizontal plane. Oppositely, the TPMS unit cell exhibits self-supported characteristics, which gives it a higher degree of freedom in geometric modeling [13]. We designed three types of TPMS porous structures using mathematical methods. The surface equations of the TPMS unit cell (gyroid, primitive, and I-WP) were input into the modeling program to construct the corresponding geometric surface mesh model. The obtained mesh surface model was converted into a Nurbs multi-surface model and used as a cutting plane to divide the unit cube volume and obtain the unit cell. It is possible to freely adjust where the unit cube is divided by offsetting the split surface, thereby constructing the TPMS porous unit cells with different relative densities. The gyroid, primitive, and I-WP surfaces can be expressed by the following implicit formulas (1)–(3) [27]:

(1) $$F_{G}(x,y,z)=\cos(x)\sin(y)+\cos(y)\sin(z)+\cos(z)\sin(x),$$

(2) $$F_{P}(x,y,z)=\cos(x)+\cos(y)+\cos(z),$$

(3) $$F_{IWP}(x,y,z)=2\cos(x)\cos(y)+2\cos(y)\cos(z)+2\cos(z)\cos(x)-\cos(2x)\cos(2y)\cos(2z),$$

**Figure 2:**
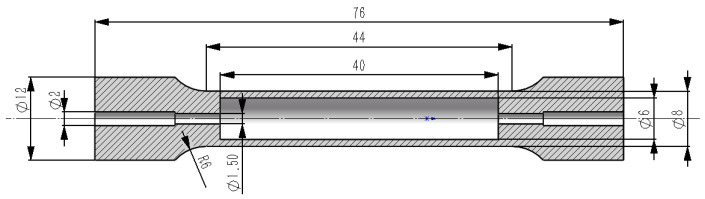
The cross-sectional figure of the tensile test specimen. The corresponding dimensions are marked in mm. The hatched region is the cross-sectional view of the test specimen shell and the blank region in the middle is the filling area after the lattice unit cells array.

All of the designed unit cells (Table 1) were 1.5 mm × 1.5 mm × 1.5 mm in size. To effectively eliminate the effect that size has on the structural performance of the unit cell, the planar array of the unit cells was set to 4 × 4 for this study [15], resulting in a section size of 6 mm × 6 mm. Furthermore, the wall thickness of the tensile test specimen shell was designed to be 1 mm, making the total diameter of the tensile test specimen 8 mm. According to ISO 6892-1 [28], a proportional relationship exists between the gauge length and the diameter of the tensile test specimen: *L*_0_ = 5*d*_0_. Therefore, we set the porous unit cell array to 4 × 4 × 27 and intersected the obtained porous block with a cylinder measuring *Φ 8* × *40* using a Boolean operation. The porous cylinder was combined with the test specimen shell to obtain the geometric model that was used for the SLM manufacturing. The dimensions of the remaining parts of the test specimen are marked in Figure 2. The holes measuring *Φ 2* and *Φ 1.5* are powder outlet holes for cleaning the unmelted powder remaining after the SLM manufacturing process.

### 2.2. Experimental Equipment, Materials, and Processes

**Figure 3:**
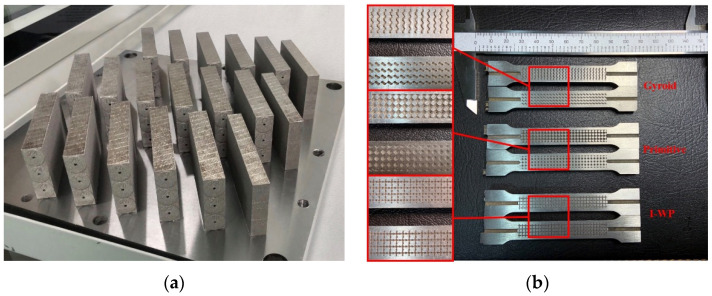
The porous test specimens manufactured by SLM are shown (**a**) and the internal structure of the test specimens (**b**) [27].

Six types of porous structures with various relative densities and the original test specimen samples were manufactured via SLM manufacturing technology. To ensure sufficient experimental samples, three samples were prepared for each type, using Inconel 718 superalloy. The manufacturing platform was an EP-M250 machine (Beijing e-plus 3D Tech. Co., Beijing, China). To improve the efficiency of the SLM manufacturing, a tree-like cumulative manufacturing method was adopted, as shown in Figure 3a, where the test specimen manufactured in the previous layer was used as the platform for laser printing in the next layer. This eliminates manual substrate replacement, which greatly reduces the total manufacturing time. After the SLM manufacturing was completed, the layers were cut by wire cutting machine (Taizhou Sihai CNC Machine Tool Factory., Taizhou, China), and the test specimens were processed by the lathe (Bochi Machine Tool Group Co., LTD., Baoji, China) according to the size in Figure 2. The internal structures of the test specimens are shown in Figure 3b [27]. The SLM processing parameters are shown in Table 2. All specimens were prepared under an argon atmosphere with the oxygen content below 100 ppm.

### 2.3. Mechanical Testing and Microstructure Analysis

A uniaxial tensile test was performed on the test specimens using a CMT-5105 universal testing device (Zhuhai SUST Electrical Equipment Co., Ltd., Zhuhai, China) equipped with a 100 kN load cell. The test was based on a metallic materials tensile test method (ISO 6892-1) [28] and evaluated the tensile properties of the original test specimen and six porous test specimens filled with lattice structure at five different relative densities. The deformation rate was set to 0.6 mm/min for all tensile tests. The mechanical property data of the original and the porous test specimens were obtained experimentally; prior experiment commencement the gauge length of the test specimen was marked and the diameter was measured. We added an extensometer with a gauge of 25 mm during the test to accurately determine the deformation in the gauge segment of the test specimen and to subsequently acquire the precise Young’s modulus through data processing. The extensometer was removed when the deformation reached 0.2 mm. This was done to keep the extensometer from being damaged during the fracture vibration of the test specimen and to ensure the complete extraction of the elastic segment deformation data. After the tests, the elongation of the test specimens and the diameter of the fracture location were measured. The experimental results, such as the percentages of elongation and reduced area of the test specimens, were then calculated. Micromorphology analysis was carried out on the fractured surface by SEM (TESCAN, Shanghai, China) to study the effect of multi-scale organizational structures (microstructure and topological design) on the mechanical properties of the test specimens.

## 3. Results

### 3.1. Lightweight Design Effect Analysis of the Test Specimens

During the tensile tests, only the parallel and transition segments of the test specimen, collectively referred to as the stressed segment, bore the tensile force. To accurately illustrate the overall lightweight effect of the stressed segment after the combination of the lattice structure and the solid shell, the stressed segment was recorded as the mass reduction object and the mass of the clamping ends at both ends of the test specimen was ignored. The design mass of the stressed segment in the original test specimen was 23.0397 g, and the design mass reduction value and percentage mass reduction of the stressed segment of the porous test specimens filled with different relative density lattice (40–80%) are shown in Table 3.

### 3.2. Mechanical Behavior

**Figure 4:**
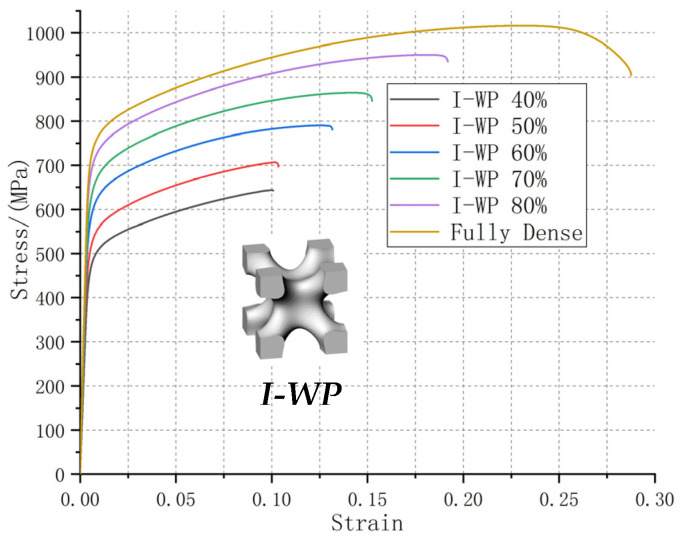
The stress-strain curves of the I-WP TPMS porous test specimen with 40–80% relative densities. The geometric model of the I-WP unit cell is provided. The stress-strain curve of the original test specimen was used as a reference.

**Figure 5:**
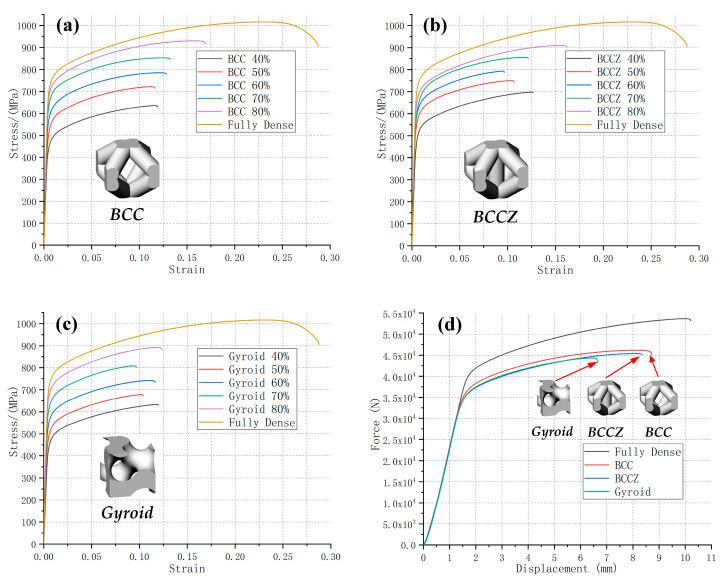
The stress-strain curves of metal lattice test specimens (**a**) BCC and (**b**) BCCZ, as well as (**c**) the gyriod TPMS porous test specimen, all with 40–80% relative densities. The corresponding geometric model of the unit cell is provided. The stress-strain curve of the original test specimen was used as a reference. (**d**) The force-displacement curves of metal lattice test specimens BCC and BCCZ, as well as the gyriod TPMS porous test specimen, with a relative density of 80%. The force-displacement curve of the original test specimen was used as a reference.

**Figure 6:**
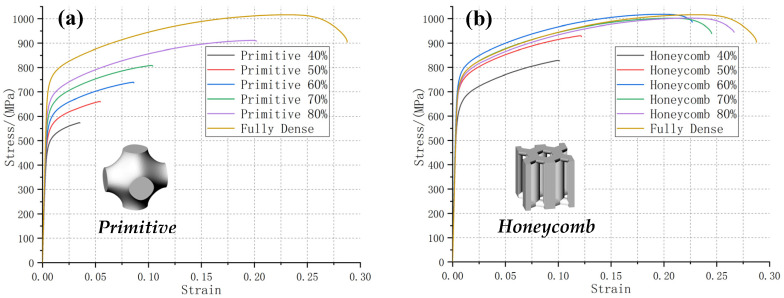
The stress-strain curves of (**a**) the primitive TPMS porous test specimen and (**b**) the honeycomb metal lattice test specimen, all with 40–80% relative densities. The corresponding geometric model of the unit cell is provided. The stress-strain curve of the original test specimen was used as a reference.

Figure 4, Figure 5 and Figure 6 clearly show that the stress-strain curve is steep at first and then tends to be gentle, indicating that all lattice test specimens first passed through the stage of elastic deformation and subsequently entered the stage of deformation strengthening. After reaching the tensile strength limit, the curve showed a downward trend and subsequently fracture occurred. The necking phenomenon was not obvious, especially in lattice test specimens with lower relative density. They broke immediately after reaching the tensile strength limit. The height and extension length of the stress-strain curve indicate the tensile strength and toughness of the test specimen, respectively. Typically, the strength and toughness of the porous test specimen should increase linearly as the relative density increases. However, our experimental results do not completely align with the design expectations. The six types of porous test specimens can be roughly divided into three categories based on their strength and toughness behaviors in relation to relative density: those whose changes in strength and toughness with relative density conforms to the law of linearity, which is in line with the design expectation; those whose change in strength with relative density meets the law of linearity, while their toughness remains constant; and those whose strength becomes constant upon the relative density reaching a certain level, while their toughness increases abruptly with increasing relative density.

#### 3.2.1. Strength and Toughness Conforming to the Law of Linear Change

The changes in tensile strength and the elongation percentage of the I-WP TPMS porous test specimen with different relative densities (40–80%) were observed from the stress-strain curves (Figure 4). Toughness indicates the ability of a material to absorb energy during plastic deformation and fracture. The greater is the tensile elongation percentage at break, the better is the toughness of the material. The changes in the strength and toughness of the I-WP test specimen agree with the design expectations, where the tensile strength and the elongation percentage linearly increased with relative density. As the relative density decreased, the difference in tensile strength between the I-WP test specimen and that of the reference specimen increased: 9.49% (8I), 17.37% (7I), 23.56% (6I), 32.18% (5I), and 38.47% (4I). The difference in elongation percentage also increased: 32.29% (8I), 47.03% (7I), 55.57% (6I), 65.52% (5I), and 67.15% (4I). All above experimental data were obtained by analyzing the stress-strain curves of the I-WP test specimens under different relative densities in Figure 4. The elongation percentage of the I-WP test specimens were significantly lower than that of the original model. This is reflected by all the test specimens with BCC, BCCZ, and gyroid structures, but only the low relative density test specimens of the primitive (≤70%) and honeycomb (≤50%) structures (Figure 5 and Figure 6).

#### 3.2.2. Basically Consistent in Toughness

The changes in tensile strength and elongation percentage of test specimens with the BCC and BCCZ metal lattice structures and those with the gyriod TPMS structure, with different relative densities (40–80%), were observed (Figure 5a–c). The tensile strength of all three types gradually increased with relative density, showing a proportional, linear relationship. The elongation percentage was maintained at a substantially uniform level for each relative density: 11–16%, 9–15%, and 9–12% for the BCC, BCCZ, and gyroid test specimens, respectively. The BCCZ test specimen exhibited the greatest strength among the three types of test specimen, while the BCC test specimen displayed the best toughness. The force-displacement curves of BCC, BCCZ and gyroid test specimen (Figure 5d) have the same changing trend, and the analysis of the curves shows that their load capacity is basically the same.

#### 3.2.3. Abrupt Changes in Toughness

The changes in tensile strength and elongation percentage of the primitive TPMS porous test specimen and the honeycomb metal lattice test specimen, with the same, varying relative densities (40–80%), were then analyzed (Figure 6a,b). The tensile strength of the primitive test specimens linearly increased with relative density, and the elongation percentages showed the same linear trend, but only when the relative density was less than 70%. When the relative density reached 80%, the elongation percentage of the test specimen sharply increased. The primitive test specimen also exhibited a similar, sudden increase in toughness between 70% and 80% relative density. The difference in elongation percentage between the primitive test specimens and that of the reference specimen was 21.12% at 8P and 64.81% at 7P.

The honeycomb test specimen was the most unique out of all six types, whose strength and toughness changed via different trends at the different relative densities. The strength level of the honeycomb test specimen was the highest amongst all six types. When the relative density was lower than 60%, its tensile strength adhered to linear change laws that were similar to those the other five test specimens followed, and, at relative densities greater than 60%, its tensile strength remained constant at a high level. The difference in tensile strength between the honeycomb test specimens and that of the reference was 2.77% at 6H, 2.48% at 7H, and 3.29% at 8H. Similar to the primitive test specimen, the honeycomb test specimen also showed a sudden increase in toughness, where the elongation percentage rose sharply between 50% and 60% relative density. At relative densities greater than 60%, the toughness of the honeycomb test specimen remained constant at a high level. The difference in the elongation percentage between the honeycomb test specimen and that of the reference was 16.93% at 6H, 9.24% at 7H, and 5.86% at 8H. The sudden changes in toughness that were observed amongst the test specimens are worthy of further investigation, and the analysis of this phenomenon is detailed in Section 4.3 and Section 4.4.

## 4. Discussion

### 4.1. Prediction Model of Equivalent Young’s Modulus

The relation between Young’s modulus and the relative density of a porous structure can be described by the Gibson-Ashby model [29]:

(4) $$E/E_{s}=C\times {\left(\rho /\rho_{s}\right)}^{n}$$

where E and ρ represent the Young’s modulus and density, respectively, and subscript s represents the substrate material property. The tensile experimental results show that the Young’s modulus of SLM Inconel 718 superalloy is about 199.51 GPa ($E_{s}$). The porous structural constant (*C* and *n*) are calculated via the Gibson-Ashby model, which represents the mechanical property of the porous structure with different relative densities. The Young’s modulus of the test specimen was determined by linearly fitting the linear elastic part of the stress-strain curve to obtain its slope. Three tensile tests were carried out for each porous test specimen with each relative density. The Young’s modulus of each porous test specimen was calculated by averaging the results of the three tests. The corresponding averaging values of E are substituted into Equation (4) to fit the Gibson-Ashby model. The porous characteristic parameters obtained via fitting are shown in Table 4.

The best data fit was obtained for the gyroid TPMS porous structure, and the correlation coefficients R^2^ was 0.99. For a porous structure, the relative density (*ρ*/*ρ_s_*) is the most critical structural parameter that influences the Young’s modulus.

**Figure 7:**
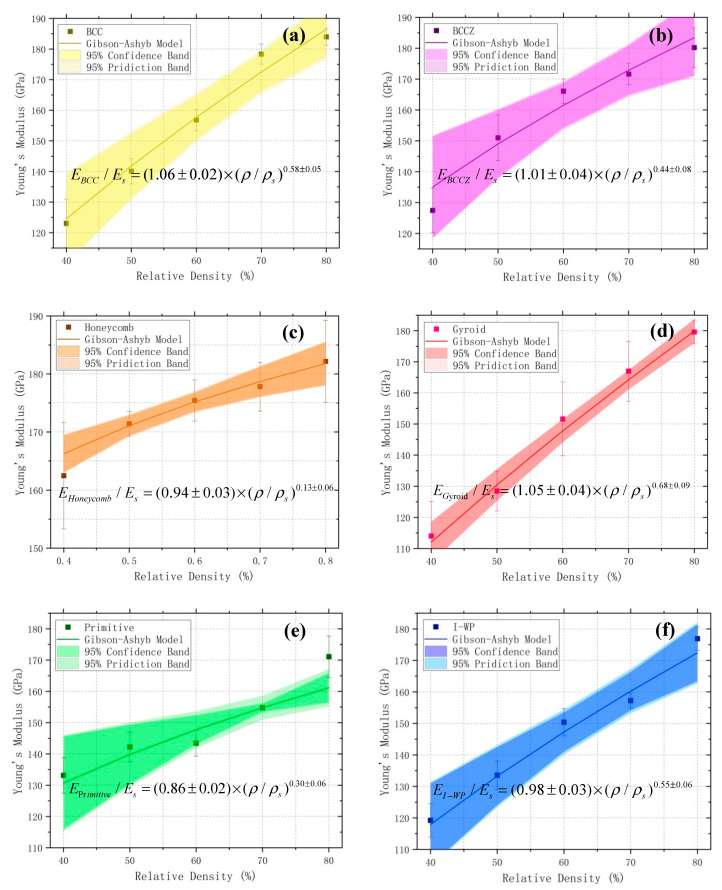
Gibson-Ashby model fitting curves for test specimens with the following lattice structures: (**a**) BCC; (**b**) BCCZ; (**c**) honeycomb; (**d**) gyroid; (**e**) primitive; and (**f**) I-WP. The darker and lighter colored bands in each figure represent the 95% confidence band and 95% prediction band, respectively.

We observed from the fitting curves and their corresponding equations (Figure 7) that the Young’s modulus of the SLM-manufactured porous test specimens increased with relative density, which is consistent with expectations for porous materials in the Gibson-Ashby model. Moreover, differences can be observed between the estimated Young’s modulus obtained via experiments and the Gibson-Ashby model. These differences may be attributed to the residual stress inherent in SLM-manufactured parts, as well as the waviness and roughness of strut surfaces [12].

**Figure 8:**
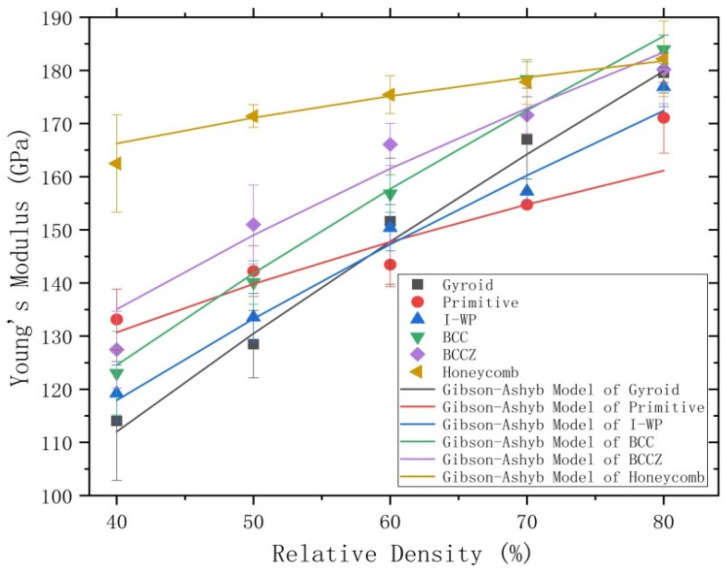
Comparison of the Young’s modulus of the six types lattice structures fitted by the Gibson-Ashby model.

When comparing the Young’s modulus of the different porous test specimens (Figure 8), the differences are more obvious at lower relative densities, but then the values become more similar as the relative density increases. All six porous test specimens can be divided into two groups, according to changes in their Young’s modulus with relative density. The Young’s modulus of the BCC, BCCZ, gyroid, and I-WP porous test specimens all showed steep slopes as relative density increased, indicating that their structural rigidity is greatly affected by changes in relative density. However, the Young’s modulus of the honeycomb and primitive porous test specimens exhibited relatively gentle slopes as relative density increased.

Earlier research has demonstrated that the porous structural constant n in the Gibson-Ashby model is commonly used to assess the deformation behavior of lattice structures [30,31]. The honeycomb porous test specimen has presented superior stiffness to those of the other test specimens and it seems to be the least influenced by varying relative density, which is due to its lower n exponent value of 0.13 ± 0.06. Furthermore, the honeycomb structure exhibits stretching-dominated deformation behavior. Similar behavior is exhibited by the primitive structure. On the other hand, the gyroid porous structures exhibits bending-dominated deformation behavior and the gyroid test specimen displays poor stiffness and is severely influenced by changes in relative density due to its higher n exponent value of 0.68 ± 0.09.

Upon comparing the changes in the Young’s modulus of the BCC and the BCCZ porous test specimens, we found that BCCZ exhibits more gentle changes than BCC, indicating that the n exponent value of BCCZ (0.44 ± 0.08) is lower than that of BCC (0.58 ± 0.05). This is a result of the vertical struts within the BCCZ structure, which makes its deformation behavior dominated by bending and stretching and less affected by changes in relative density. The I-WP porous structure is also known as the TPMS-based BCC structure [32] and the n exponent value of the I-WP test specimen is approximately equal to that of the BCC test specimen. Moreover, the I-WP structure exhibits bending-dominated deformation behavior similar to that of the gyroid structure. The above analysis was based on results of Ashby et al. [30,31,33], indicating that the test specimens filled with bending-dominated porous structures have lower strength and stiffness than those of filled with stretching-dominated structures and are more susceptible to changes in relative density.

### 4.2. Comparison of Mechanical Properties

**Figure 9:**
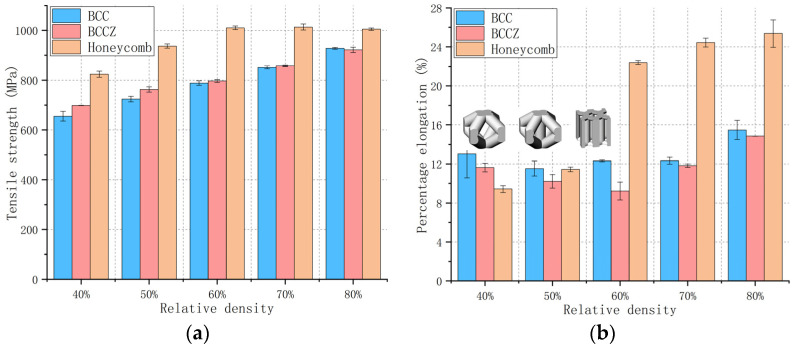
Histograms of (**a**) the tensile strength and (**b**) elongation percentage of the BCC, BCCZ, and honeycomb metal lattice test specimens with various relative densities. The x-axis denotes relative density: 40–80%, respectively. The blue bars represent BCC, the red denotes BCCZ, and the orange denotes honeycomb.

We compared the tensile strength and the elongation percentage of the BCC, BCCZ and honeycomb test specimens at different relative densities. Figure 9a shows that the tensile strength of the honeycomb test specimens was the highest under all relative density. In addition, Table 5 shows that the tensile strength of the honeycomb test specimen is higher than all three TPMS test specimens under each relative density. This is because there are six vertical struts in the honeycomb unit cell that are consistent with the direction of the tensile force, which has the best unidirectional force bearing characteristics.

Besides, the tensile strength of the BCCZ test specimens was significantly greater than that of the BCC test specimens when the relative densities is less than 60%, as shown in Figure 9a. However, this difference in tensile strength became less pronounced as the relative density increased. This is because the struts in both structures are thin when the relative density is small, and the vertical struts of the BCCZ structure are what dictate its overall tensile performance. As the relative density increases, the struts become thicker and the support from the vertical struts in the BCCZ structure is significantly weakened, resulting in the strength of the two structures becoming more similar.

Furthermore, Figure 9b also shows that the elongation percentage of the BCCZ porous test specimens was always lower than that of the BCC test specimens. This is due to the existence of the vertical struts in the BCCZ structure, which ensures that the strut diameter of the BCCZ unit cell is always smaller than that of the BCC under the same relative density. Additionally, the contact area between the struts and the unit cell is smaller in the BCCZ structure than in the BCC structure, making the BCCZ more prone to deformation and damage. This shows that, although some lattice properties can be improved by redesigning the unit cell structure, other properties may also be affected. This should be comprehensively considered during the design process.

### 4.3. Analysis of Unit Cell Structure Characteristics

**Figure 10:**
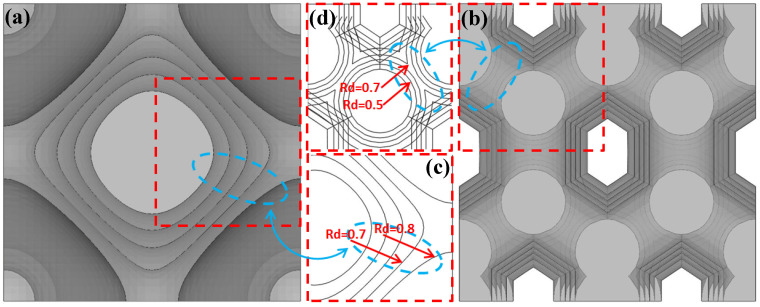
Superimposed effect diagrams of: (**a**) the top view in rendering mode of the primitive unit cell under the five relative densities; (**b**) the top view in the rendering mode of the honeycomb unit cell under the five relative densities; (**c**) the partial top view of the primitive unit cell in wireframe mode (red arrows indicate the unit cell top profiles with relative densities of 70% (Rd = 0.7) and 80% (Rd = 0.8)); and (**d**) the partial top view of the honeycomb unit cell in wireframe mode (red arrows mark the top profiles of the vertical pillar inside the honeycomb unit cell with relative densities of 50% (Rd = 0.5) and 70% (Rd = 0.7)).

The reason for the sudden changes in toughness observed for the primitive and honeycomb porous test specimens could be found by analyzing their unit cell structural characteristics (Figure 10). It can be seen that the profile of the primitive unit cell with 70% relative density (Rd = 0.7) is not spliced together with the adjacent unit cells, and there are still interpenetrating pores between the unit cells after the array. During the stretching process, the connection surfaces between the unit cells were not large enough to provide effective support to each other to resist the plastic deformation; therefore, the test specimen was prone to failure and damage, and exhibited lower toughness level as a result. The structural profile for the primitive unit cell with 80% relative density (Rd = 0.8) was extended to the boundary of the unit cube. The pores of the unit cell after the array were self-closing and the unit cells were completely connected to each other. The plastic deformation generated during the stretching process was effectively distributed to the whole stress section of the test specimen, thereby significantly improving its toughness.

It can be seen that the profile of the vertical pillars inside the honeycomb unit cell with 70% relative density (Rd = 0.7) have been extended and spliced together, allowing the unit cell to more effectively resist plastic deformation. Moreover, the vertical pillars of the adjacent unit cells connect to make a whole after the array, further improving the ability of the test specimen to resist plastic deformation. As a result, the toughness level of this test specimen is close to that of the original test specimen. When the relative density of the unit cell was 60%, the vertical pillars are not yet connected to each other (Figure 10d). Despite this, the gap between adjacent pillars is already extremely small. We considered that when the test specimen underwent slight plastic deformation due to stretching the vertical pillars could make contact and provide one another with enough support to resist plastic deformation, resulting in significantly improved toughness. The honeycomb test specimens with relative densities less than 60% exhibited similar load-bearing characteristics to those of the primitive test specimens with relative densities less than 70%, and their overall toughness was low.

### 4.4. Fracture Analysis

**Figure 11:**
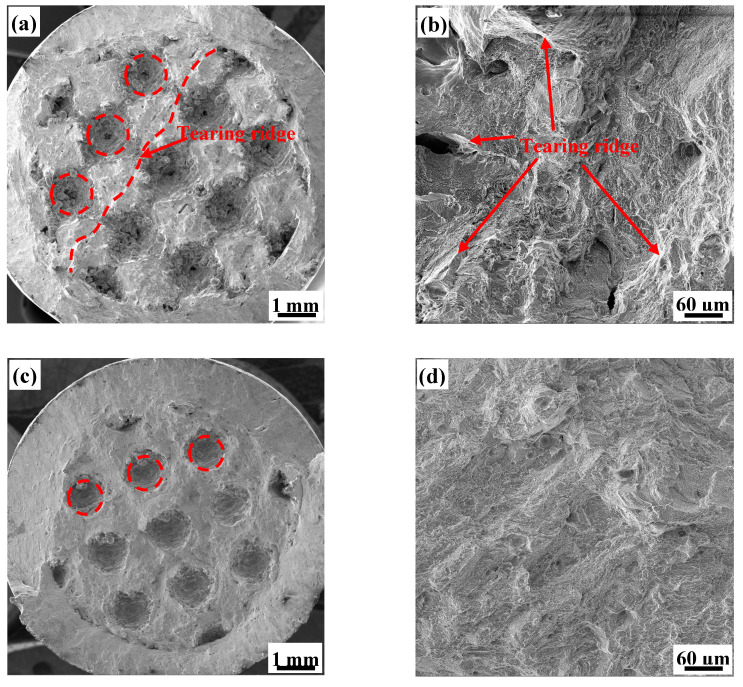
Scanning electron microscopy (SEM) images of: (**a**) the complete fracture morphology of 7P (dotted line depicts a tearing ridge); (**b**) the local fracture micromorphology at high-magnification of 7P; (**c**) the complete fracture morphology of 8P; and (**d**) the local fracture micromorphology at high-magnification of 8P.

**Figure 12:**
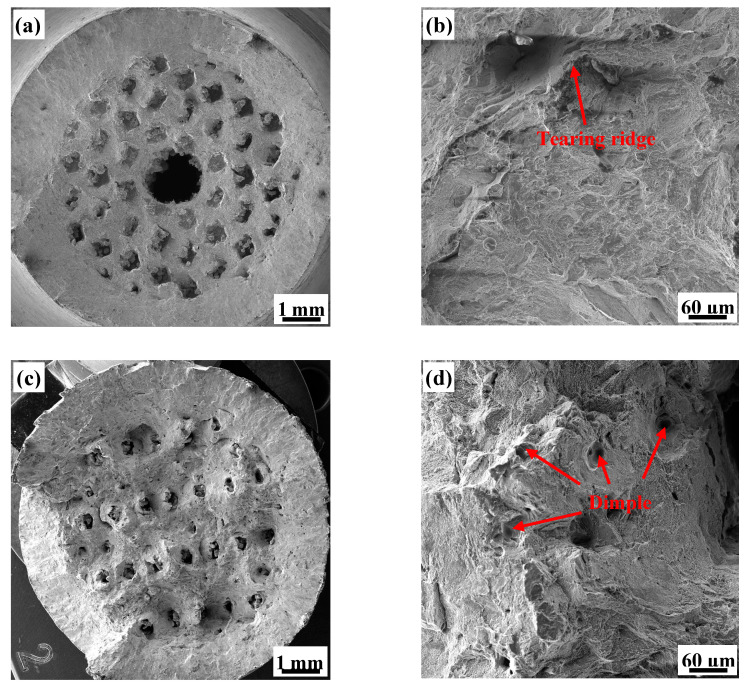
SEM images of: (**a**) the complete fracture morphology of 5H; (**b**) the local fracture micromorphology at high-magnification of 5H; (**c**) the complete fracture morphology of 7H; and (**d**) the local fracture micromorphology at high-magnification of 7H.

To further investigate the microscopic mechanism of the sudden changes in toughness exhibited by the 7P, 8P, 5H, and 7H test specimens, fracture surfaces of the porous structures after the tensile tests were observed by scanning electron microscopy (SEM) (Figure 11 and Figure 12). The micromorphology of each test specimen showed microfeatures of quasi-cleavage fractures, but there are obvious differences in the local characteristics of each test specimen. The quasi-cleavage fracture is a transitional fracture mechanism between the cleavage fracture and ductile fracture. Its micromorphology is characterized by short and curved river-like patterns with few tributaries, which are mixed with different numbers of tearing rigids and dimples [34,35].

The overall fracture surface of the primitive porous test specimen was divided into three regions (Figure 11a,c): the “spherical” pore area formed by unit cell arrays in the central region; the unit cell fracture surface; and the shear lip, which formed an included angle of 45° with the applied tensile stress. The pore area of the 7P structure is obviously larger than that of the 8P structure. We predominantly focused on the fracture surface of the unit cell. We observed an obvious tearing ridge on the 7P fracture surface, while the fracture surface of 8P was relatively flat. The micromorphology of the fracture surface (Figure 11b,d) is mainly river-like patterns while that of 7P displays many tearing ridges in different directions, which were formed by the rapid instability and fracture of the test specimen under normal stress without undergoing stress redistribution. This is a typical fracture characteristic of brittle materials; therefore, the 7P test specimen exhibits obvious brittleness when it breaks. On the other hand, the 8P test specimen has a large bearing area due to the splicing of the unit cells. This allows its tensile stress to be evenly distributed along the whole force section, providing better toughness than that of 7P.

The overall fracture surface of the honeycomb porous test specimen (Figure 12a,c) is similar to that of the primitive, as it also can be divided into three areas. The pore area has an irregular shape due to the deformation of the test specimen. The fracture surface of the 5H unit cell is relatively flat (Figure 12a,b), with its microscopic morphology being dominated by river-like patterns and presenting few tearing ridges. On the other hand, the fracture surface of the 7H unit cell is uneven, but the transition is smooth and there are a large number of tiny holes on the fracture surface (Figure 12c). With higher magnification (Figure 12d), we observed that these tiny holes are actually large dimples (tens of microns in size) in which smaller dimples are nestled. This phenomenon shows that the 7H test specimen experienced a more complicated stress evolution process during fracturing. When the stress breaks the tensile limit the unit body quickly releases the deformation to absorb the energy of the breaking front, which terminates failure or directs it elsewhere. As a result, the microscopic morphology constitutes a large number of uniformly distributed dimples on the unit cell fracture surface, indicating that 7H has excellent toughness.

The test specimen and experimental designs employed this study can be further optimized. When designing the test specimen, the experimental marking can be carried out on the solid shell, which is also used as the carrier held by the extensometer. However, its existence affects the accuracy of the test results of the lattice structure mechanical properties to a certain extent. In future research, the shell thickness should be reduced as much as possible. The complicated stress environment endured by the heat-resistant, load-bearing components during actual application subjects them to bending, torsion and vibration, as well as fatigue, tensile, and compressive loads. Therefore, regarding experimental design and optimizing the lattice topology, it is obviously not enough to only evaluate the unidirectional, load-bearing characteristics of the lattice. We can add other experiments to test bending and torsion characteristics, which will more comprehensively evaluate the mechanical properties of the lattice structure. Furthermore, experiments testing mechanical properties in high-temperature environments and fatigue characteristics can be combined with heat treatment processes for further research. Building off of another idea [36], we can also apply complex loads to the unit cube and use topological optimization theory to conduct innovative lattice configuration design research. In addition, we can reconstruct the model of the SLM-manufactured lattice structure using micro-CT scanning. This would be based on the component design and finite element simulation of mechanical properties that could be carried out and obtain more realistic simulation results, and could be used as a reference for future, related research.

## 5. Conclusions

This study investigated issues involving appropriate lattice selection for lattice topology optimization of heat-resistant, load-bearing structures. Using Inconel 718 superalloy and SLM technology, an original test specimen was fabricated along with six others: porous test specimens with BCC, BCCZ, and honeycomb metal lattices and TPMS test specimens with gyroid, primitive, and I-WP lattices. Each was designed at five different relative densities: 40%, 50%, 60%, 70%, and 80%. Static tensile tests were carried out in triplicate on each test specimens to evaluate their mechanical properties, where abrupt changes in toughness were observed for the primitive and the honeycomb test specimens at 70~80% and 50~60% relative density, respectively. The Gibson-Ashby model was used to nonlinearly fit the Young’s modulus of each lattice test specimen. SEM was employed to analyze the micromorphology of the fracture surface of the test specimens with an abrupt change in toughness.

1. Ignoring the mass of the clamping ends at both ends of the test specimen, the mass reduction percentage of the porous test specimens at the different relative densities were as follows: 24.30% (40%), 20.25% (50%), 16.20% (60%), 12.15% (70%) and 8.10% (80%).
2. The tensile strength of the BCC, BCCZ, gyroid, primitive, and I-WP test specimens showed proportional, linear relationships with relative density. However, the tensile strength of the honeycomb porous test specimen was the highest and remained unchanged when the relative density was higher than 60%. The difference in tensile strength between the honeycomb porous test specimen and that of the original specimen was 2.77% at 6H, 2.48% at 7H, and 3.29% at 8H.
3. The elongation percentage of the I-WP test specimen showed a proportional, linear relationship with the relative density, while that of the BCC, BCCZ, and gyroid test specimens remained substantially uniform over ranges of 11–16%, 9–15% and 9–12%, respectively, at each relative density.
4. Difference in the Young’s modulus of the different porous test specimens were more pronounced at lower relative densities, but the values become more similar as the relative density increased. The honeycomb porous test specimen also possessed the best stiffness among the test specimens evaluated and was least influenced by varying relative density. According to the range of the n exponent value and the deformation laws of the porous structures, we concluded that the porous structure exhibited a stretching-dominated deformation behavior when n < 0.3, a bending-dominated deformation behavior when n > 0.55, and a stretching-bending-dominated deformation behavior when 0.3 < n < 0.55.

## Figures and Tables

**Table 1:** Body-centered cubic (BCC), BCC with Z-struts (BCCZ), honeycomb, gyroid, primitive, and I-WP unit cells with 40–80% relative density.

Porosity Structure	40%	50%	60%	70%	80%
BCC	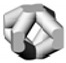	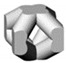	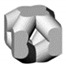	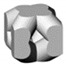	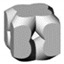
BCCZ	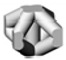	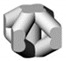	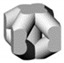	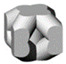	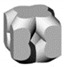
Honeycomb	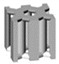	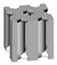	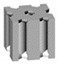	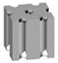	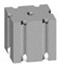
Gyroid	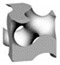	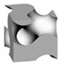	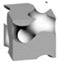	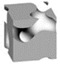	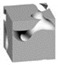
Primitive	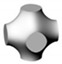	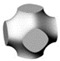	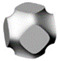	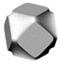	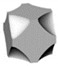
I-WP	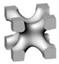	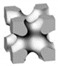	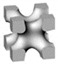	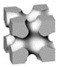	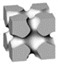

**Table 2:** Selective laser melting (SLM) parameters used to produce porous tensile test specimens for mechanical testing.

SLM Parameter	
Laser power	285 W
Laser scan speed	960 mm/s
Laser area width	5 mm
Laser hatch spacing	110 μm
Power deposition thickness	30 μm
Spot diameter	90 μm

**Table 3:** Design mass reduction value and percentage mass reduction of the stressed segment of the porous test specimen.

Relative Density	Mass Reduction Value/g	Percentage Mass Reduction
40%	5.5915	24.30%
50%	4.6596	20.25%
60%	3.7277	16.20%
70%	2.7958	12.15%
80%	1.8638	8.10%

**Table 4:** Porous structural constant (*C* and *n*) and the fitted correlation coefficient R^2^ for each lattice structure.

Lattice Type	*C*	*n*	Correlation Coefficient
BCC	1.06 ± 0.02	1.06 ± 0.02	0.96
BCCZ	1.01 ± 0.04	1.01 ± 0.04	0.90
Honeycomb	0.94 ± 0.03	0.94 ± 0.03	0.94
Gyroid	1.05 ± 0.04	1.05 ± 0.04	0.99
Primitive	0.86 ± 0.02	0.86 ± 0.02	0.85
I-WP	0.98 ± 0.03	0.98 ± 0.03	0.95

**Table 5:** Summary of the tensile strength ($\sigma_{b}$/MPa) of the honeycomb, gyroid, primitive, and I-WP porous tensile test specimens under different relative densities (40~80%).

Lattice Type	40%	50%	60%	70%	80%
Honeycomb	824.4 ± 12.6	937.0 ± 8.4	1010.6 ± 7.2	1013.6 ± 12.2	1005.1 ± 4.6
Gyroid	888.6 ± 7.3	691.1 ± 11.8	626.1 ± 16.3	749.0 ± 6.3	816.1 ± 10.6
Primitive	577.4 ± 3.2	661.4 ± 3.3	738.3 ± 9.9	806.2 ± 8.4	919.4 ± 14.0
I-WP	639.5 ± 8.7	704.9 ± 3.4	794.5 ± 3.6	858.80 ± 9.6	940.7 ± 12.1
